# Necrological

**Published:** 1880-06

**Authors:** 


### NECROLOGICAL,

"Death is the crown of life:
Were death denied, poor man would live in vain."

Keener.—Suddenly, in this City, on the 21st of May, Dr. William H. Keener, in the 59th year of his age, at the residence of his sister, Mrs. Dr. A. F. Dulin, Sr., No. 83 Monument street. Deceased was born in this City in 1821, and was the son of the late Dr. David Keener, for many years the superintending chemist at the old Canton Copper Works. He was educated at the old Baltimore College and spent many years abroad. The day before his death he complained of a pain in the head, but his indisposttion was not regarded by his friends as serious.

